# A case report of septic shock and splenic abscess formation secondary to gastric band erosion: A rare complication of laparoscopic adjustable gastric banding

**DOI:** 10.1016/j.ijscr.2020.03.015

**Published:** 2020-04-01

**Authors:** Victoria Lu, Harsh Kanhere

**Affiliations:** Upper Gastro-intestinal Surgical Unit, The Queen Elizabeth Hospital, Adelaide, South Australia, Australia

**Keywords:** Laparoscopic adjustable gastric band, Gastric band erosion, Splenic abscess, Gastric band complication, Bariatric surgery

## Abstract

**Introduction:**

With the rising burden of obesity, bariatric surgery is becoming more common as a treatment option. Laparoscopic adjustable gastric banding (LAGB) is considered safe and effective and is a popular procedure in Australia. However there are recognised complications to be aware of such as band erosion which can lead to infection and abscess formation.

**Presentation of case:**

A 59-year-old caucasian female presented with fevers, rigors and feeling generally unwell. She was previously fit and well with her only past medical history being LAGB 14 years prior. Clinically the patient was in septic shock and required intensive care admission for inotropic support. On investigations the CRP was 227 and abdominal computed tomography (CT) revealed a splenic abscess. Follow up upper gastrointestinal endoscopy diagnosed an eroded gastric band in the stomach. The patient proceeded to laparoscopy, a gastrotomy was performed and the band was removed. The splenic abscess was concurrently drained and the patient treated with an extended course of intravenous and oral antibiotics.

**Discussion:**

Band erosion is a rare but serious complications of LAGB surgery along with band slippage, pouch dilatation and abscess formation. Patients are often asymptomatic making early diagnosis difficult. Upper gastrointestinal endoscopy is used to locate the band and recommended treatment is band removal via laparoscopy or laparotomy.

**Conclusion:**

Band erosion should be suspected in patients with a history of LAGB presenting with nonspecific symptoms such as abdominal pain or fevers. This case also highlights the importance of appropriate patient follow up post operatively and counselling of operative risks and long-term complications.

## Highlights

• General Surgery.
• Bariatric Surgery.
• Splenic Abscess.
• Gastric Banding.
• Upper Gastrointestinal Surgery.

## 1 Introduction

Obesity is a rising issue contributing to the major health burden of chronic disease worldwide. The WHO estimates that worldwide more than 1.9 billion adults are either overweight or obese [1]. In addition to lifestyle changes (dietary modification, behaviour change, increased physical activity), bariatric surgery should be considered in a proportion of severely obese patients [2]. Bariatric surgery has been performed since the 1950s with procedures such as jejuno-ileal bypass and gastric stapling procedures (Roux Y gastric bypass). The development of laparoscopic approaches in the last two decades such as LAGB has shown improved safety with better patient outcomes and acceptable weight loss [3]. In Australia, LAGB surgery is the commonest bariatric procedure performed, however it is not without complications. These include band erosion, pouch dilatation and band slippage [4]. It is important to recognise such complications given the high number of gastric bands that exist in the community.

We report a rare case of splenic abscess secondary to gastric band erosion occurring 14 years after insertion, in a patient who presented unwell with non specific symptoms. This case was complicated by the formation of a splenic abscess and contributed to sepsis. This was managed with laparoscopic removal of the band and drainage of the splenic abscess. The work of this case report has been reported in line with the SCARE 2018 criteria [5].

## 2 Presentation of case

**Fig. 1:**
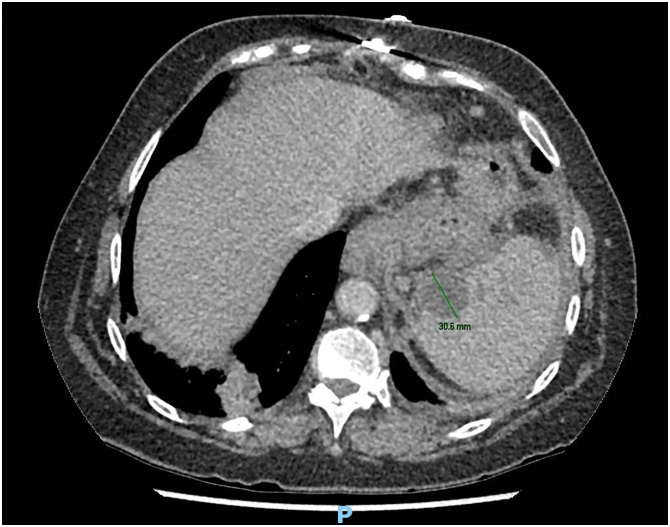
Abdominal computed tomography showing an axial view of the splenic abscess.

**Fig. 2:**
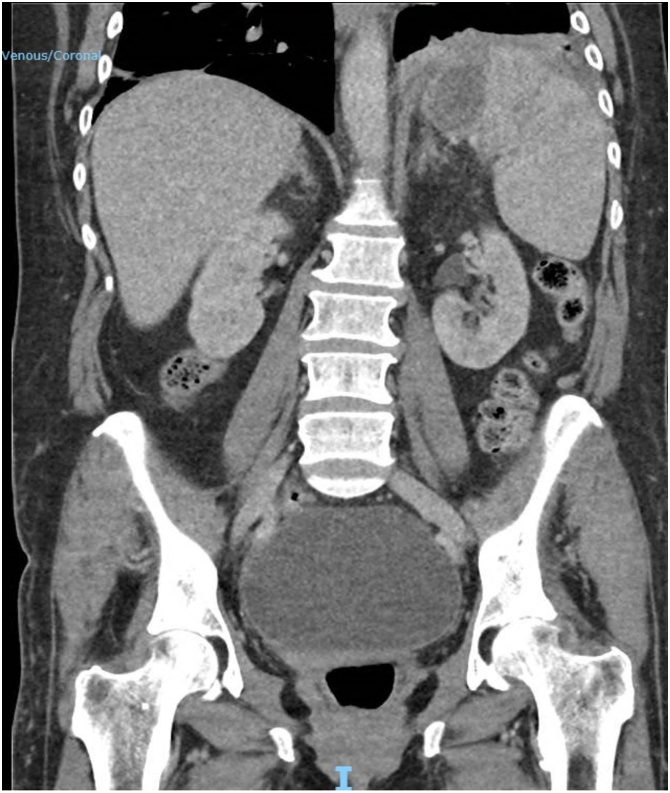
Abdominal computed tomography showing a coronal view of the splenic abscess and incidental large ovarian cyst.

**Fig. 3:**
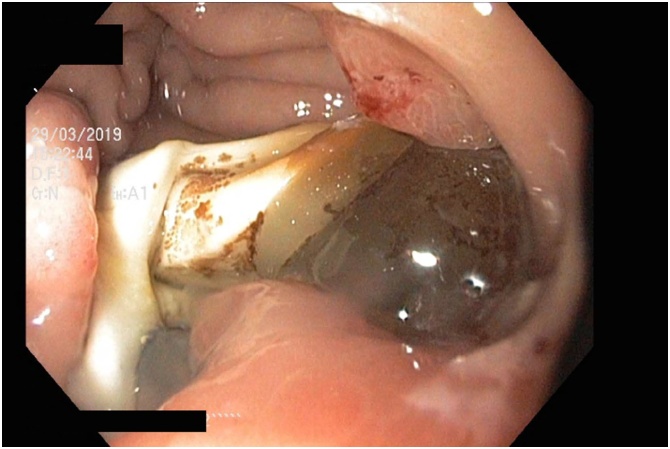
Endoscopic image of the eroded gastric band in the stomach.

**Fig. 4:**
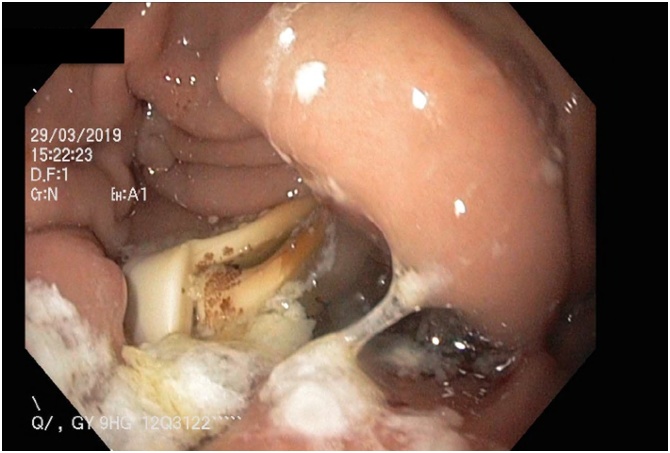
Endoscopic image of the eroded gastric band in the stomach.

A 59 year old Caucasian female presented to the emergency department with a 3-day history of fevers, rigors and feeling generally unwell. There was no history of abdominal pain, nausea, or vomiting. Her past medical history was significant for laparoscopic adjustable gastric banding (LAGB) 14 years prior. On examination the patient was hypotensive, tachycardic, febrile and was given a preliminary diagnosis of septic shock. She was subsequently resuscitated and admitted to intensive care and required inotropic support. She was investigated for possible sources of sepsis; however, all preliminary testing was unremarkable except for a markedly raised C reactive protein level of 227 mg/L. Abdominal computed tomography (CT) was performed and revealed a 30 mm intra-splenic collection and an incidental large ovarian cyst (Fig. 1, Fig. 2). An endoscopy was then conducted, which identified an eroded gastric band in the stomach (Fig. 3, Fig. 4).

A diagnosis of sepsis secondary to an infected and eroded gastric band was made. Broad spectrum intravenous antibiotics were commended and a decision was made to proceed with operative management under the Upper-gastrointestinal Surgery Unit. A laparoscopy was initially performed, however the band was unable to be visualised within the peritoneal cavity. A gastrotomy was subsequently performed and the was band identified and successfully removed. The splenic abscess was concurrently drained intra-operatively. The gynaecology surgical team also performed a bilateral salpingo-oophorectomy to remove the large ovarian cyst.

Post-operatively the patient recovered well. Blood cultures demonstrated polymicrobial growth consistent with gastrointestinal flora. This was managed with an extended course of intravenous antibiotics and the patient was later discharged on a course of oral antibiotics and followed up in surgical outpatient clinic.

## 3 Discussion

Gastric band erosion is a recognised but rare complication of LAGB where the band erodes through the stomach wall over time and into the gastric lumen [6]. The incidence of band erosion is reported to be between 0.5–3.8% [7]. Other complications of LAGBs include port malfunction, band erosion, band slippage, band leakage and gastroesophageal reflux disease [8]. Predisposing factors to erosion include chronic tissue damage, such as excessive band filling and surgeon experience. Studies report a correlation between rates of band erosion and surgeon experience with the annual risk of occurrence highest in the first two years of surgical practice and decreasing over time [9]. Diagnosis can be challenging as most patients are asymptomatic or present with nonspecific symptoms such as abdominal pain and fevers, therefore a high index of clinical suspicion is required to diagnose band erosion [10]. Diagnosis is usually made via upper gastrointestinal endoscopy. Studies show nether CT nor contrast swallows are sensitive enough for definitive diagnosis, although can be useful in the initial workup [3]. Once diagnosed, band erosion always requires removal of the band either laparoscopically or via laparotomy. In addition, LAGBs can predispose to intraabdominal abscesses and, if present, should be drained and treated with appropriate antimicrobial therapy [10].

## 4 Conclusion

Gastric band erosion should be considered in patients with a history of gastric banding presenting with sepsis. Diagnosis can be made via upper gastrointestinal endoscopy to localise the band and imaging such as CT can be utilised to identify any surrounding complications such as abscess formation. This case supports the recommended treatment for gastric band erosion which is surgical removal of the gastric band [11]. This is an important complication for surgeons to be aware of as early diagnosis is key to prevent significant morbidity and mortality.

## Funding

No funding.

## Ethical approval

Ethics approval not required.

## Consent

Informed verbal and written signed consent for publishing of case and all images has been obtained from the patient.

## Author contribution

Victoria Lu, MBBS: Writing – original draft and review.

Harsh Kanhere, MS: Writing – review and editing, supervision.

## Registration of research studies

NA.

## Guarantor

Victoria Lu.

## Provenance and peer review

Not commissioned, externally peer reviewed.

## Declaration of Competing Interest

No conflicts of interest.
